# Modified arthroscopic suture‐button Latarjet procedure for recurrent anterior shoulder instability: 9‐Year minimum follow‐up

**DOI:** 10.1002/ksa.70305

**Published:** 2026-01-26

**Authors:** Wei Lu, Daqiang Liang, Shengwu Yang, Yan Liu, Guoqing Cui, Chunwu Zhang, Shuguang Gao, Zhenhan Deng

**Affiliations:** ^1^ Department of Orthopedics Shenzhen Hospital of Southern Medical University Shenzhen Guangdong China; ^2^ Department of Sports Medicine The First Affiliated Hospital of Shenzhen University, Shenzhen Second People's Hospital Shenzhen Guangdong China; ^3^ Department of Orthopedics The First Affiliated Hospital of Wenzhou Medical University Wenzhou Zhejiang China; ^4^ Geriatrics Center The First Affiliated Hospital of Wenzhou Medical University Wenzhou Zhejiang China; ^5^ Department of Infection and Critical Care Medicine The First Affiliated Hospital of Shenzhen University, Shenzhen Second People's Hospital Shenzhen Guangdong China; ^6^ Department of Nosocomial Infection Prevention and Control The First Affiliated Hospital of Shenzhen University, Shenzhen Second People's Hospital Shenzhen Guangdong China; ^7^ Institute of Sports Medicine Peking University Third Hospital Beijing China; ^8^ Departmentof Orthopaedics, Xiangya Hospital Central South University Changsha Hunan China

**Keywords:** complications, Latarjet, remodelling, suture‐button, union

## Abstract

**Purposes:**

To evaluate the efficacy of the modified arthroscopic suture‐button (MASB) Latarjet procedure with at least 9 years of follow‐up and remodelling of the coracoid grafts (CGs).

**Methods:**

Patients who underwent the MASB Latarjet procedure between February 2013 and February 2016 were retrospectively reviewed. The clinical outcomes, complications, union and remodelling of the CGs and glenohumeral degeneration were assessed postoperatively in a follow‐up period of at least 9 years.

**Results:**

A total of 106 patients were enroled with a mean follow‐up of 110.3 ± 4.2 months. Significant improvements were observed in visual analogue scale (VAS) pain scores during motion, the American Shoulder and Elbow Surgeons (ASES), Rowe and Walch‐Duplay scores (*p* < 0.001), with no significant range of motion (ROM) limitations at final follow‐up. The complication rate was 8.5%, including two cases of instability, one requiring revision surgery. In the en‐face sagittal view, 86.8% of grafts were excellently or well‐positioned, and 89.6% were flush with the glenoid in the axial view. CG‐glenoid union occurred in 99.1% of cases, restoring 99.5 ± 1.4% of the PFC, with no evident arthropathy at final follow‐up.

**Conclusion:**

The MASB Latarjet provided durable shoulder stability and high patient satisfaction at 9‐year follow‐up, with low complications, minimal ROM loss and no evident glenohumeral degeneration.

**Level of Evidence:**

Level IV, case series.

**Abbreviations**
3D CT: three‐dimensional computed tomography
ANOVA: analysis of variance
ASES: the American Shoulder and Elbow Surgeons
BMI: body mass index
CG: coracoid graft
FRF: flexible rivet fixation
GBL: glenoid bone loss
GDA: glenoid defect area
ISIS: the Instability Severity Index Score
MASB: modified arthroscopic suture‐button
OA: osteoarthritis
PFC: perfect fitting circle
POD: postoperative day
POM: postoperative month
POW: postoperative week
POY: postoperative year
RASI: recurrent anterior shoulder instability
ROM: range of motion
RPP: return to previous performance
RTP: return to play
VAS: visual analogue scale

## INTRODUCTION

The Latarjet procedure has been proven to be an effective and reliable procedure for the treatment of recurrent anterior shoulder instability (RASI), especially for patients with glenoid bone loss (GBL) or bipolar bone loss (humeral head and glenoid) or anterior irreparable capsular ligament injury, and to serve as a salvage procedure in the setting of failed primary soft tissue repair [7, 33]. In 2019, a modified arthroscopic suture‐button (MASB) Latarjet procedure was described based on the technique proposed by Boileau et al. [1], which achieved excellent outcomes with few complications and no detection of degenerative changes at 3‐year follow‐up [37]. It was found that mild coracoid graft (CG) absorption occurred within six months after the surgery, primarily at the edges and outside the glenoid perfect fitting circle (PFC), a concept first proposed by Sugaya et al as the ‘outer fitting circle’ [32]. If the healed CG failed to cover the original PFC at early postoperative period, it will tend to expand outward and eventually achieve full coverage of the PFC. This phenomenon was particularly evident in 12% of cases where the CG was fixed too superiorly or inferiorly as seen in three‐dimensional computed tomography (3D CT) sagittal views. Notably, during the study period, cases with significantly laterally positioned CG, as shown in transverse CT scans, did not lead to joint arthropathy and eventually remodelled to a glenoid arc surface concentric with the humeral head [37].

The popularity of suture‐button fixation, Latarjet, belong to the flexible rivet fixation (FRF), for treatment of RASI with large GBL continues to grow [2, 4, 31]. However, there have been few studies reporting the long‐term results of the FRF Latarjet procedure. The function of the shoulder joint may decrease over time, and it was reported that the prevalence of glenohumeral osteoarthritis (OA) after the traditional screw fixation Latarjet procedure ranges from 49% to 71% in the long‐term follow‐up [26].

Although the MASB Latarjet procedure has achieved good results at 3‐year follow‐up without obvious signs of glenohumeral OA [37], its long‐term results remain unknown. The current study aimed to analyse the clinical effects of the MASB Latarjet procedure, investigate remodelling of the CGs after performing the FRF technique and assess clinical impact. We hypothesized that the MASB technique would result in excellent long‐term outcomes, with further remodelling and union of the CGs and minimal degenerative changes.

## METHODS

### Study design

This study was a case series from the same cohort of patients as our prior 3‐year follow‐up study [37]. It included patients who underwent the MASB Latarjet procedure for RASI between February 2013 and February 2016, with a minimum 9‐year clinical and radiographic follow‐up to evaluate the long‐term outcomes of the procedure. The ethical approval for this study was granted by the ethical committee of our institute (No. 20210722001‐FS02), and all included patients provided written consent.

### Patients selection

The glenoid defect area (GDA) was calculated based on preoperative 3D CT, and the depth of the Hill‐Sachs lesion was measured on axial and coronal CT scans [32]. The Instability Severity Index Score (ISIS) was calculated per established criteria: age <20 years (2 points); participation in competitive sports (2 points) or contact/collision sports (1 point); shoulder hyperlaxity (positive sulcus sign, 1 point); radiographic Hill‐Sachs lesion (2 points) and significant GBL (2 points). Scores ≥4 indicate high failure risk, favouring Latarjet [27].

Inclusion criteria were: (1) RASI with GDA > 20%, (2) RASI with 15% < GDA < 20% and ISIS > 6, (3) RASI with 10% < GDA < 15% and competitive sports participation and (4) revision for Bankart repair failure. Exclusion criteria were: (1) epilepsy, (2) incomplete follow‐up data or loss to follow‐up and (3) prior shoulder surgery other than Bankart repair.

### Surgical technique and rehabilitation

All surgical procedures were performed by a senior chief physician. The detailed MASB Latarjet surgical technique as well as rehabilitation protocol were introduced in previous publications [20, 36, 37]. Briefly, the CG and the conjoint tendon were prepared via a 2.5 cm mini‐open incision. The coracoacromial ligament and part of the pectoralis minor were cut 1 cm from the coracoid border. A 2 cm CG was osteotomized using an oscillating saw. Two bone tunnels were drilled along the graft's axis. A high‐strength suture was passed through the proximal tunnel for traction, and three sutures were threaded through a suture button via the distal tunnel. Anterior, anterolateral and posterior portals were established. The glenoid was marked at the 4 o'clock position, and the subscapularis muscle was split about 1.5 cm from back to front. A glenoid tunnel was drilled using a customized guide, and the graft was pulled into the glenohumeral joint via the guide suture. A second Endobutton was placed behind the glenoid, secured with a Tennessee knot. A knotless anchor was fixed on the glenoid through the proximal tunnel to prevent rotation.

### Outcome measures

The primary outcome was the American Shoulder and Elbow Surgeons (ASES) score at 9‐year follow‐up, chosen to assess overall shoulder function and patient satisfaction, directly aligning with the study objective (evaluating long‐term efficacy of MASB Latarjet) and hypothesis (excellent long‐term outcomes with sustained remodelling and minimal degeneration). Secondary clinical outcomes included visual analogue scale (VAS) pain scores (during motion), Rowe and Walch‐Duplay scores, active shoulder range of motion (ROM; forward flexion, abduction, external/internal rotation at side and 90° abduction), return to play (RTP; any sports participation) and return to previous performance (RPP; same/higher pre‐injury level), recurrence rate (subluxation as subjective anterior translation with spontaneous reduction [14] or dislocation requiring third‐party reduction [15]), complications and reoperations [10, 38]. All were assessed preoperatively and at postoperative years (POY) 3 and 9.

Radiographic outcomes included: (1) CG position (vertical in en‐face sagittal view: excellent >75% GDA coverage, good 50%–75%, fair 25%–49% and poor <25% [23]; horizontal in axial view: flush, medial >5 mm and lateral >3 mm [18]); (2) The CG‐glenoid interface union was assessed using imaging studies performed at POY 1. Union of the CG was graded as bony union, fibrotic union (radiolucent zone <5 mm), or migration (radiolucent zone >5 mm) [14]. (3) Humeral head degeneration was evaluated using the standard described by Samilson and Prieto: (1) normal; (2) mild (osteophytes <3 mm on the humeral head); (3) moderate (osteophytes between 3 and 7 mm) and (4) severe (osteophytes >7 mm) [28]. Imaging (radiography and 3D CT) was performed preoperatively, postoperative day (POD) 0, postoperative month (POM) 6, POY 1, 2, 3–5 and 6–9. Evaluations were blinded and independent from two orthopaedic fellows.

### Sample size calculation

Prior to patient enrolment, an a priori power analysis was conducted using G*Power 3.1. Based on the results of our previous 3‐year cohort [37], which showed an average improvement of approximately 10 points in the ASES score with a standard deviation of 15, a minimum of 34 patients would be required to detect such a difference with a two‐sided *α* of 0.05 and 80% power. Considering a 20% potential loss to follow‐up, the target sample size was 42 patients.

### Statistical analysis

All data were expressed as mean and standard deviation. Normality of the continuous variables was assessed using the Shapiro–Wilk test, and all variables were confirmed to follow a normal distribution. A randomized block analysis of variance (ANOVA) was used to analyse the response variable at preoperative, POY 3 and POY 9 follow‐ups while controlling for individual differences. When the overall ANOVA indicated significance, post hoc pairwise comparisons were conducted with Bonferroni adjustment to identify specific differences between preoperative and follow‐up measures. The SPSS Statistics software package (version 20.0; IBM) was used for all the statistical analyses, and *p* < 0.05 was considered statistically significant.

## RESULTS

### Patient data

A total of 133 patients were enroled in this study, and 27 patients were lost to follow‐up or excluded because of incomplete follow‐up data. Ultimately, 106 patients (106 shoulders) were enroled, comprising 91 males and 15 female patients.

The characteristics of the patients who returned for follow‐up visits and radiological evaluations are shown in Table 1. The mean follow‐up time was 110.3 ± 4.2 months. The mean age was 25.4 ± 4.7 years (range, 18–36 years). All patients were diagnosed with RASI due to trauma or other reasons. The mean number of dislocations before they received surgery was 8.8 ± 5.3. The mean GDA was 19.3 ± 5.7% (range, 6%–28%). In addition, 55 patients had GDA > 20%, 26 had GDA of 16%–20% with ISIS > 6, 15 had GDA 11%–15% with competitive sports level, and 10 had failed Bankart repair. In addition, 104 patients had Hill‐Sachs lesions to differing extents (Table 1).

**Table 1:** General information of the included patients.^a^

Parameter	Value
Age at surgery, mean ± SD (range), y	25.4 ± 4.7 (range, 18–36)
Gender, male/female	91/15
Number of dislocations	8.8 ± 5.3
Body mass index, kg/m^2^	24.3 ± 5.1
GDA, mean ± SD, %	19.3 ± 5.7%
>20%	55 (51.9)
15%–20%	26 (24.5)
10%–14%	15 (14.2)
<10%	10 (9.4)
Bankart failure	10
Hill‐Sachs injury	104

Abbreviations: GDA, glenoid defect area; SD, standard deviation.

^a^ Data are shown as *n* (%) unless otherwise indicated.

### Subjective pain

The VAS scores for pain during motion decreased from a mean of 3.3 ± 1.0 (range, 1–6) preoperatively to 1.2 ± 0.7 (range, 0–3) at POY3, and 0.1 ± 0.4 (range, 0–2) at POY9. Compared with preoperative status, the improvements in pain during motion of both postoperative time points were statistically significant (*p* < 0.001, Table 2).

**Table 2:** Functional results preoperatively and at final follow‐up.

Parameter	Preoperative	POY 3	POY 9	*p* Value
VAS for pain (during motion), mean ± SD (range)	3.3 ± 1.2	1.2 ± 0.7	0.1 ± 0.4	<0.001
Range of motion, deg				
Forward flexion	173.7 ± 15.5	169.9 ± 14.5	174.6 ± 11.2	<0.001
Abduction	127.2 ± 13.2	126.7 ± 15.2	128.3 ± 14.6	<0.001
External rotation at the side	78.6 ± 13.1	73.1 ± 9.2	78.0 ± 10.8	<0.001
External rotation at 90° of abduction	78.7 ± 14.0	75.9 ± 12.3	77.8 ± 12.7	0.001
Internal rotation at 90° of abduction	66.4 ± 12.0	63.7 ± 12.7	66.0 ± 12.2	0.011
ASES score	79.2 ± 14.7	95.2 ± 5.1	95.3 ± 4.3	<0.001
Rowe score	41.5 ± 10.2	94.1 ± 2.6	94.3 ± 2.9	<0.001
Walch‐Duplay score	68.3 ± 10.6	94.8 ± 3.1	95.0 ± 3.2	<0.001

Abbreviations: ASES, the American Shoulder and Elbow Surgeons; POY, postoperative year; SD, standard deviation; VAS, visual analogue scale.

### Range of motion

The mean forward flexion, abduction, external rotation at the side, external rotation at 90° of abduction and internal rotation at 90° of abduction were 173.7° ± 15.5°, 127.2° ± 13.2°, 78.6° ± 13.1°, 78.7° ± 14.0°, 66.4° ± 12.0° preoperatively; 169.9° ± 14.5°, 126.7° ± 15.2°, 73.1° ± 9.2°, 75.9° ± 12.3°, 63.7° ± 12.7° at POY 3 and 174.6° ± 11.2°, 128.3° ± 14.6°, 78.0° ± 10.8°, 77.8° ± 12.7°, 66.0° ± 12.2° at POY 9, respectively. The shoulder ROM significantly decreased at the POY 3 compared to the baseline preoperative measures (*p* < 0.05). However, by the POY 9, ROM had been restored to levels comparable to baseline (*p* = 0.202 for forward flexion, *p* = 0.082 for abduction, *p* = 0.246 for external rotation at the side, *p* = 0.069 for external rotation at 90° of abduction, and *p* = 0.171 for internal rotation at 90° of abduction, Table 2).

### RTP and subjective scores

At the last follow‐up, 87.7% of patients (93/106) were still participating in sports: 66.0% (70/106) returned to the same or higher level, and 23 patients (21.7%) changed their sports practice; 13 patients (12.0%) completely ceased sports. Significant improvements were observed in postoperative ASES scores, Rowe scores, and Walch‐Duplay scores: 79.2 ± 14.7, 41.5 ± 10.2, 68.3 ± 10.6 preoperatively; 95.2 ± 5.1, 94.1 ± 2.6, 94.8 ± 3.1 at POY 3; and 95.3 ± 4.3, 94.3 ± 2.9, 95.0 ± 3.2 at POY 9, respectively. However, there were no significant differences between POY 3 and POY 9 for the ASES score (*p* = 0.594), Rowe score (*p* = 0.279) and Walch‐Duplay score (*p* = 0.416, Table 2).

### Recurrence of instability

Among the two shoulders (1.9%) with a recurrence of instability, one had a true shoulder dislocation at POW 5 and one had subluxation at POY 4, and both instability recurrences after surgery were traumatic. The former showed no recurrence after conservative treatment, and the latter patient underwent a reoperation. Both of them were stable at the last follow‐up.

### Complications and reoperations

The overall complication rate was 8.5% (9/106) at the last follow‐up, including two patients for the recurrence of instability mentioned at ‘Recurrence of instability’, and one of the two instability underwent a reoperation. The management and outcome of all complications are summarized in Table 3.

**Table 3:** Individual description of complications and treatment at the last follow‐up (9/106 patients, 8.5%).

Patients number	Sex	Age at surgery, y	Time of complications	Previous surgery	Complications	Definitive treatment
1	Male	23	During surgery	None	CG fracture	RPP at POY 1.5 after conservative rehabilitation
2	Male	32	POD 0	None	Endobutton misplacement	RPP at POM 6 after conservative rehabilitation
3	Male	26	POW 1	None	Lateral forearm paraesthesia due to musculocutaneous nerve injury	Peripheral nerve stimulation and oral mecobalamin were given and healed at POW 6.
4	Female	32	POW 2	None	Surgical incision fat liquefaction	Wound dressing change and healed at POW 7.
5	Female	28	POW 2	None	Surgical incision fat liquefaction	Wound dressing change and healed at POW 6.
6	Male	21	POW 5	None	Redislocation	No recurrence after conservative treatment.
7	Male	36	POM 2	None	Intra‐articular infection	The infection was controlled after intra‐articular antibiotic injection every other day for 2 weeks.
8	Male	32	POM 3	None	Joint stiffness	RPP at POY 2.5 after continuous functional exercise.
9	Male	33	POM 6	Arthroscopic Bankart repair (seven metal anchors)	CG‐gelnoid soft‐union	Suture ligation and fixation are used for the secondary exploration at POY 4, and re‐union at POY 9.

Abbreviations: CG, coracoid graft; POD, postoperative day; POM, postoperative month; POW, postoperative week; POY, postoperative year; RPP, return to previous performance; RTP, return to play.

**Figure 1:**
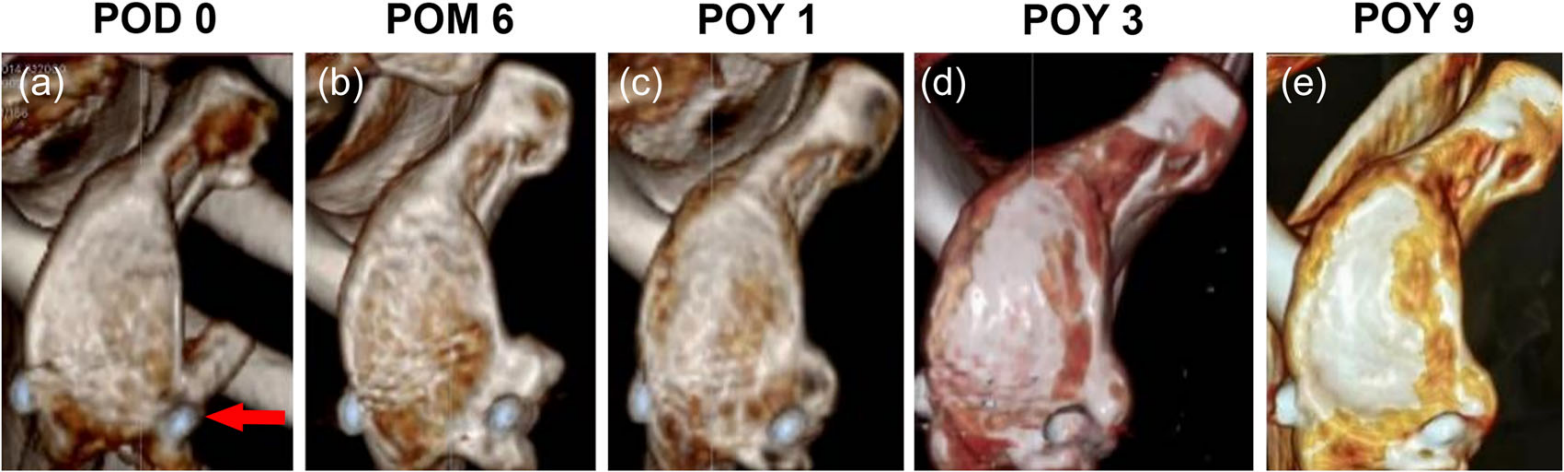
One case (No. 1) of CG fracture during the surgery (3D CT en‐face sagittal view of the glenoid). (a) The red arrow showed the CG fracture at POD 0. (b) The CG‐glenoid surface healed at POM 6. (c–e) The CG‐glenoid remodelling at different follow‐up time points. 3D, three‐dimensional; CG, coracoid graft; CT, computed tomography; POD, postoperative day; POM, postoperative month; POY, postoperative year.

There was one intraoperative complication. Although one case of CG fracture happened when using high‐strength wire to fix the knot on the Endobutton during the surgery (Patient No. 1, Figure 1a), the CG was healed at POM 6 (Figure 1b) and continuous remodelling over time (Figure 1c–e). The patient RPP until POY 1.5 after conservative rehabilitation.

**Figure 2:**
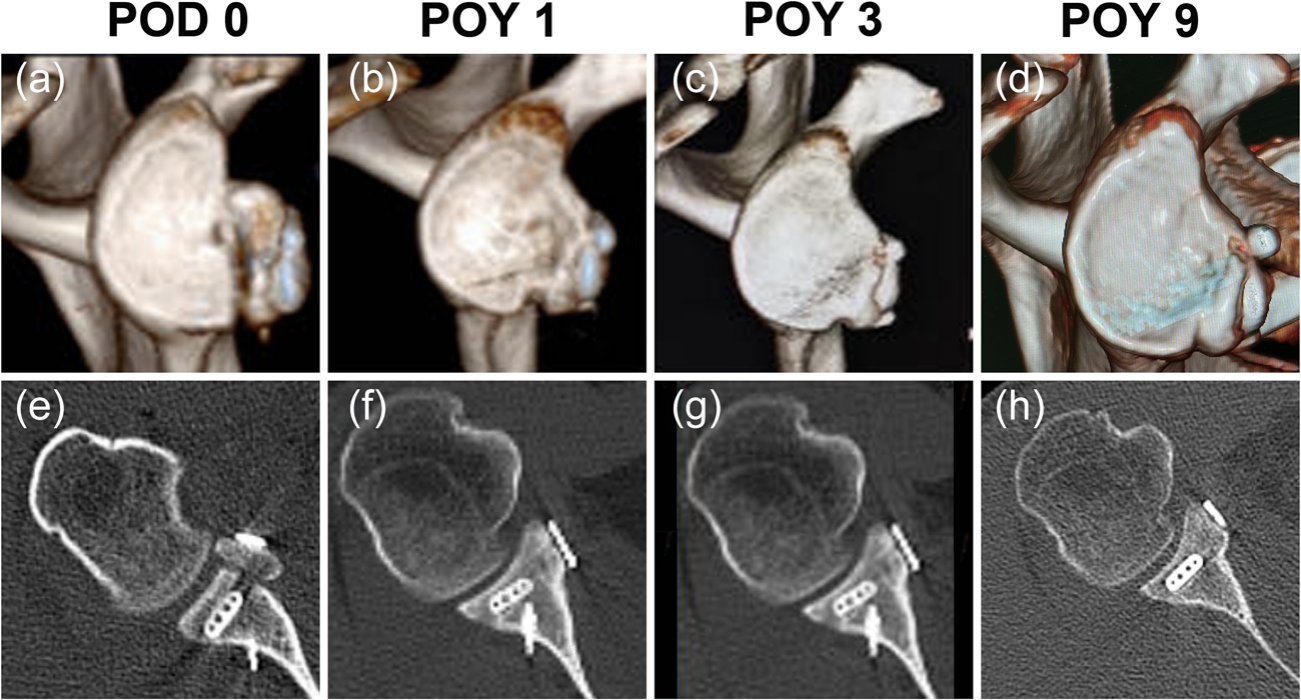
One case (No. 2) of Endobutton misplacement and remodelling of the CG‐glenoid interface of the 3D CT en face view (a–d) and the 2D CT axial view (e–h). 2D, two‐dimensional; 3D, three‐dimensional; CG, coracoid graft; CT, computed tomography; POD, postoperative day; POY, postoperative year.

One patient was found to have Endobutton misplacement immediately at postoperative CT scan, and the RPP at POM 6 after conservative rehabilitation. Although the CG was put laterally at POD 0, it healed and flushed to the glenoid surface during the follow‐up (Figure 2). One case of lateral forearm paraesthesia due to musculocutaneous nerve injury at postoperative week (POW) 1, and peripheral nerve stimulation and oral mecobalamin were given, and the patient was healed at POW 6. Two female patients suffered from surgical incision fat liquefaction at POW 2, and the wound was healed at POW 6 and 7 after continuous dressing changes. One traumatic dislocation happened at POW 5, but no recurrence after conservative treatment. One intra‐articular infection occurred at POM 2, and the infection was controlled after intra‐articular antibiotic injection every other day for 2 weeks. One joint stiffness occurred at POM 3, and the RPP at POY 2.5 after continuous functional exercise.

**Figure 3:**
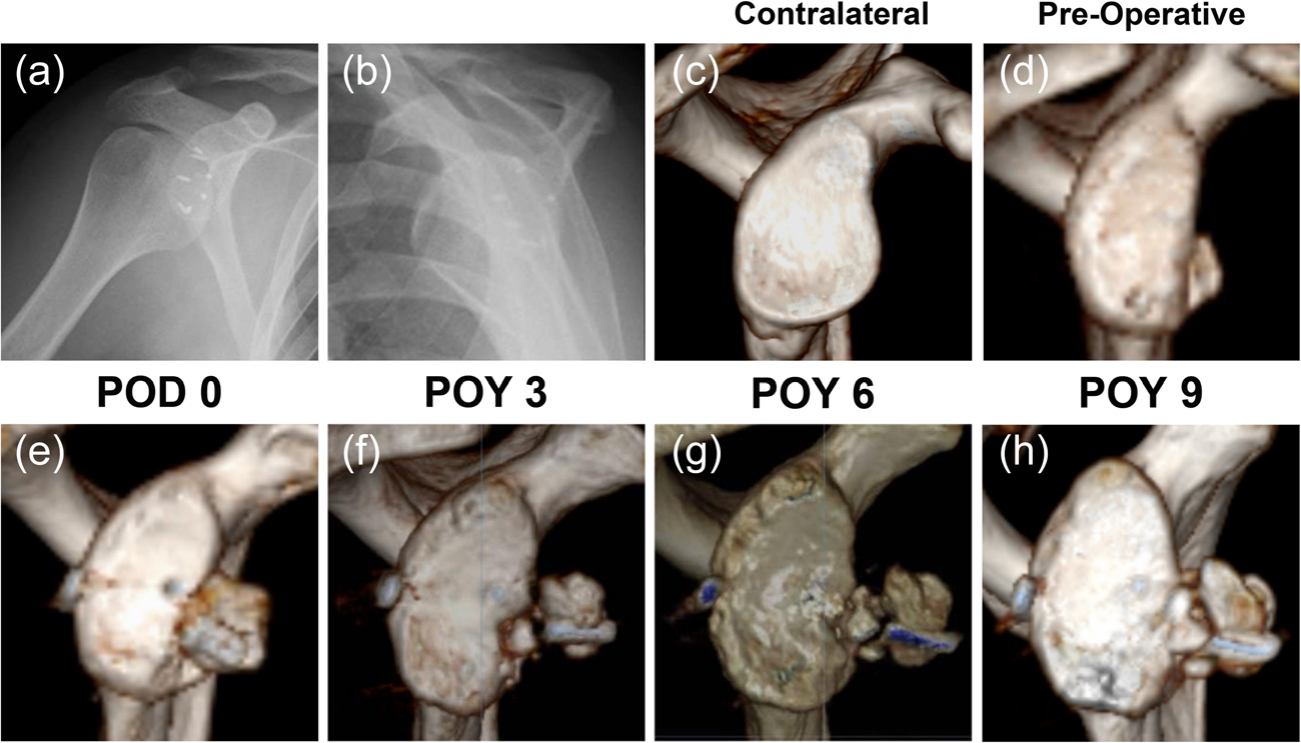
One case (No. 9) of Latarjet revision after Bankart failure of X‐ray (a,b) and 3D CT en face view (c–h). (a,b) The anteroposterior and scapular Y view X‐ray of twice Bankart repair surgeries before he undertook the Latarjet procedure. The bright signal showed the seven metal anchors left over from the previous surgery. (c) Preoperative mirror image of the normal side. (d) Preoperative injury side. (e) The CG‐glenoid interface at POD 0. (e–g) The CG‐glenoid in each follow‐up time point. 3D, three‐dimensional; CG, coracoid graft; CT, computed tomography; POD, postoperative day; POY, postoperative year.

One patient underwent two unsuccessful Bankart repair surgeries in 2011 and 2014, with a total of seven metal anchors used for fixation (Figure 3a,b). In 2016, he received a MASB Latarjet revision surgery. At POY 3, the CG was found to be obviously separated from the glenoid. During a rotator cuff repair surgery caused by traumatic subluxation at POY 4, the CG was reattached using suture ligation and fixation. Fortunately, at POY 6, the CG demonstrated a trend with glenoid union, and demonstrated progression to bony union at POY 9 (Figure 3c–g). Even so, he was satisfied with the shoulder's function, and no recurrent dislocation happened after the revision Latarjet surgery.

### Imaging assessment

#### Graft position

In the en‐face sagittal view, excellent graft positioning was achieved immediately postoperatively in 72 patients (67.9%), 20 in good position (18.9%), 10 (9.4%) in fair position and 4 (3.8%) in poor position. In the axial view, 95 (89.6%) grafts were flush to the glenoid, and 0 (0%) and 11 (10.4%) grafts were fixed medially and laterally, respectively (Table 4).

**Table 4:** Graft position in en‐face sagittal and axial views.

Location	*N* (%)
En‐face sagittal view	
Excellent ( > 75% GDA)	72 (67.9)
Good (50%–75% GDA)	20 (18.9)
Fair (25%–50% GDA)	10 (9.4)
Poor ( < 25% GDA)	4 (3.8)
Axial view	
Flush	95 (89.6)
Medial	0 (0)
Lateral	11 (10.4), 5.5 ± 2.3 mm

Abbreviation: GDA, glenoid defect area.

#### Graft union

A total of 105 (99.1%) CGs achieved bone union at the POY 1, except for one soft‐union, and he did not union at the last follow‐up (complications Patient No. 9).

#### Graft‐glenoid remodelling

**Figure 4:**
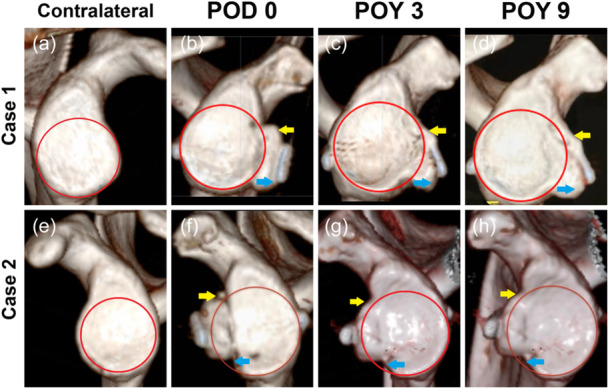
Remodelling of the CG‐glenoid interface in 3D en face view (two cases are presented). From POD 0 (b,f) to POY 3 (c,g) and POY 9 (d,h), the CG and glenoid and graft are fused and remodelled, analogous to the shape of the ‘contralateral’ intact glenoid (a,e). The red circle indicates the PFC of the glenoid. The CGs also showed that vertically remodelled, CG bone absorption occurred anterior upper outside of the PFC (yellow arrows), and the expansion at the anterior lower inside the PFC (blue arrows). 3D, three‐dimensional; CG, coracoid graft; PFC, perfect fitting circle; POD, postoperative day; POY, postoperative year.

The previous result had found all the CGs remodelled to a steady state within 2 years [37]. In the en face view, the CG and glenoid were fused and finally remodelled analogously to the shape of the intact glenoid (PFC) (Figure 4). The CG bone absorption (within 6 months) mostly occurred at the anterior upper outside of the PFC (yellow arrows), and the expansion (after 1 year) happened at the anterior lower inside the PFC (blue arrows).

**Figure 5:**
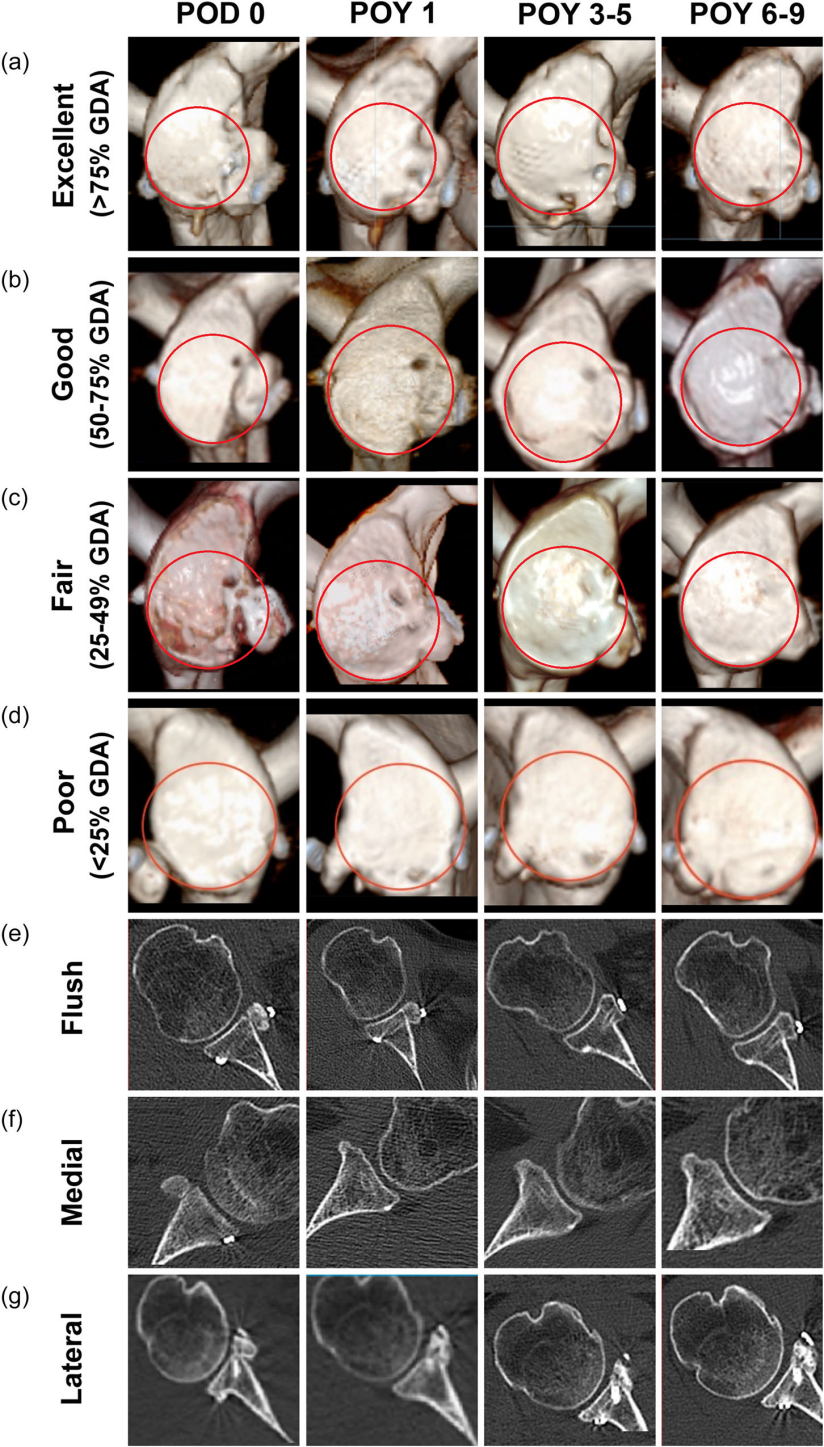
Remodelling of the CG‐glenoid interface of four representative excellent, good, fair and poor vertically positioned cases on the 3D CT en face view (a–d), and three representative flush, medial and lateral horizontal positioned cases on the 2D CT axial view (e–g) at different follow‐up time points. The red circle indicates the PFC of the glenoid. 2D, two‐dimensional; 3D, three‐dimensional; CG, coracoid graft; CT, computed tomography; GDA, glenoid defect area; PFC, perfect fitting circle; POD, postoperative day; POY, postoperative year.

The cases of excellent, good, fair and poor in the vertical position are presented in Figure 5a–d, all of the CG's remodelling tend to cover the PFC over time, even those in poor position at POD 0. For cases of flush, medial and lateral in the horizontal position, all of the CG's remodelling tended to flush to the glenoid surface (Figure 5e–g). Additionally, there was no medial CG in our cases according to Kraus TM's criteria [18]. One case that was relatively medially positioned was presented and achieved good remodelling (Figure 5f).

**Figure 6:**
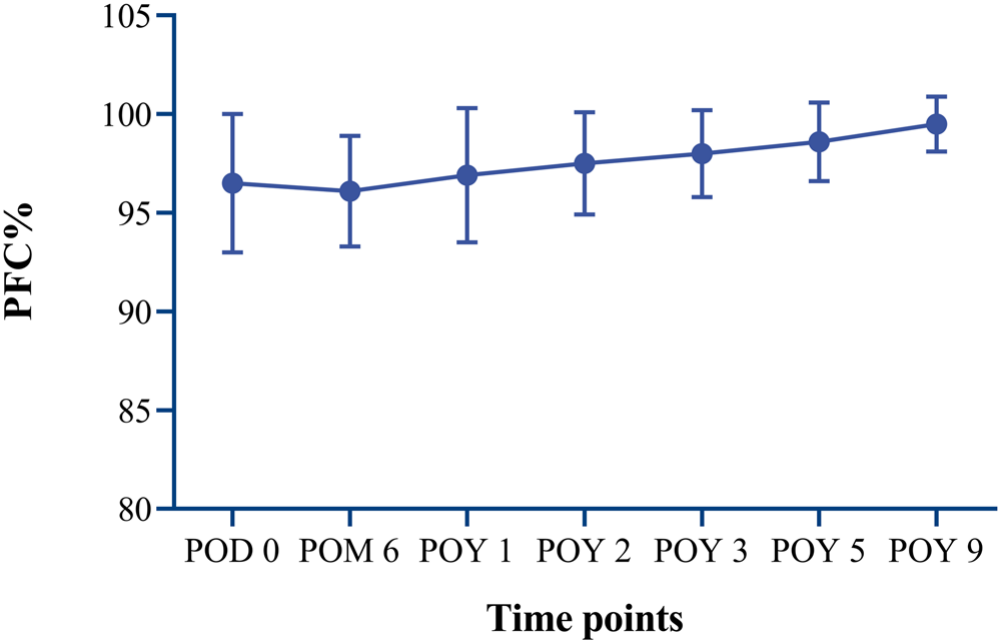
The trend of PFC restoration after MASB Latarjet procedure at different follow‐up time points. MASB, modified arthroscopic suture‐button; PFC, perfect fitting circle; POD, postoperative day; POM, postoperative month; POY, postoperative year.

The mean GDA was 19.3 ± 5.7% preoperatively. The CG restored 96.5 ± 3.5% of the PFC at POD 0, and 99.5 ± 1.4% at final follow‐up. The CG continues to grow and finally almost covers the entire PFC (Figure 6).

#### Glenohumeral joint arthropathy

**Figure 7:**
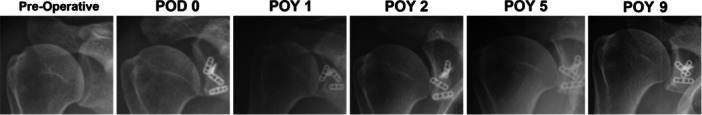
Radiographs show no degenerative changes of the shoulder joints after MASB Latarjet procedure at each follow‐up time point (one case with CG positioned laterally). CG, coracoid graft; MASB, modified arthroscopic suture‐button; POD, postoperative day; POY, postoperative year.

According to Samilson and Prieto's criteria [28], all the shoulders that underwent MASB Latarjet procedure showed no obvious degenerative changes, even those with CGs positioned laterally (Figure 7).

## DISCUSSION

The most important findings of this ≥9‐year follow‐up study, extending our prior mid‐term report [37], are as follows: (1) The MASB Latarjet procedure resulted in excellent long‐term functional outcomes and high fault tolerance, with no recurrence, a low complication rate (8.5%), and sustained ROM recovery. (2) Nearly all patients (99.1%) achieved long‐horizon CG‐glenoid bone union, with 87.7% RTP. (3) The CG‐glenoid interface exhibited sustained remodelling toward the PFC, tending to restore a concentric circle with the humeral head analogous to the original glenoid over time. (4) Mal‐positioned CGs at POD 0 did not cause glenohumeral arthropathy at 9‐year follow‐up.

In clinical evaluation, it was found that the pain and functional scores improved considerably during the follow‐up. The high level of satisfaction might be attributable to the high rate of RTP, because Warth et al found the greatest concern in patients undergoing surgery for RASI was the ability to return to sporting activity [35].

According to previous studies, the recurrence rate after the Latarjet procedure was low, ranging between 0% and 10% [3], and the recurrences mostly occurred in the POY 1–2 [11, 38]. Most of the recurrent instability events were subluxations, with recurrent dislocations comprising less than one‐third of instability events. The recurrence rate is as low as 1.9% in our case series, which might be due to high CG union rate and the ‘triple‐blocking’ mechanism of the Latarjet procedure [6]. The authors believe that this technique provides firm fixation and that no treatment is necessary if the patient remains on track after surgery, and previous literature also has similar views [8]. Therefore, no additional treatment was performed for Hill‐Sachs lesion, and no obvious sign of shoulder instability was observed at the last follow‐up.

The most common reason for revision was recurrent instability, although this occurred in less than 2% of patients. Our MASB Latarjet procedure used suture‐button for CG fixation to avoid the hardware‐related complications, such as screw breakage, loosening or screws penetrating into the joint, and so on [11]. Although only one revision happened in our study, complications remain a concern with the Latarjet procedure, given that reported data range from 11% to 30% complication rate after the Latarjet procedure in the literature [11, 24].

Osteolysis, a significant complication, occurs primarily in the anterosuperior quadrant [17]. According to a recent systematic review, suture button fixation in the Latarjet procedure has a lower bone resorption rate (10.1%–18.5%), while the rate is significantly higher in screw fixation (25.2%–47.6%) [13]. Suture‐button fixation, as used in our study, results in minimal CG absorption, with grafts expanding to restore the PFC [23, 37]. The sling mechanism may shift the humeral head's rotation centre anteriorly, promoting slight glenoid expansion. Initially malpositioned CGs remodel to a concave glenoid surface, facilitated by the flexibility of suture‐button fixation [5, 34], as supported by our recent animal study [22].

Subscapularis dysfunction and fatty degeneration after tenotomy are reported in prior studies, possibly due to large splits interfering with innervating nerves during management [29, 30]. Surgical approach modifications reduce fatty infiltration but yield significant deficits in active internal/external rotation and strength versus the contralateral shoulder [9]. In the MASB Latarjet, the subscapularis was split only 8–10 mm laterally to the glenoid, protected by a switch stick to minimize axillary nerve interference [21]. Slight ROM limitations occurred at POY 3 but recovered to preoperative levels by POY 9, which may be due to minimal subscapularis invasion and nerve protection.

Our overall complication rate was 8.5% at POY 9, avoiding the common issues noted above. Intraoperative CG fracture and postoperative joint stiffness resolved with systematic rehabilitation and exercise, enabling RTP. Surgical incision fat liquefaction occurred in two female patients with relatively high body mass index (BMI). Special attention to perioperative wound care is recommended for this population. As salvage surgery for Bankart repair failure, Patient No. 9 experienced graft separation and nonunion after the MASB procedure, but CG re‐reattachment led to glenoid union between POY 6 and 9, suggesting re‐fixation as a viable option in such MASB cases. Additionally, some degree of residual pain was found in a significant number of patients [11]. Hurley et al. considered that the residual pain may be caused by gradual degenerative changes in the joint [15]. As no obvious degenerative changes were observed in our case series, this may explain the absence of persistent pain during follow‐up, except for transient pain in specific movements during strenuous exercise in a few patients.

Arthropathy is the primary concern in long‐term follow‐up. Studies identify risk factors for progressive instability arthropathy, including older age, high‐demand sports and lateral positioning of transferred CGs relative to the glenoid rim [12, 16, 25]. A recent systematic review of 13 long‐term Latarjet studies found that over one‐quarter of patients progressed in arthropathy grade [21]. Less than 10% overall had Grade II/III arthropathy at final follow‐up. In most shoulders, degenerative changes were limited to one‐grade progression per the Samilson–Prieto classification, with only one patient receiving arthroplasty. A 22‐year screw‐fixation study reported 34.1% OA, with the author citing lateral CG overhang as the sole surgical risk factor [19]. In contrast, our 9‐year series, though relatively short, showed no obvious degenerative changes, even in laterally positioned cases. This low degeneration rate may be attributed to suture‐button fixation, which is the key advantage of FRF [23].

This study has several limitations. First, its retrospective, non‐randomized design limits the ability to establish causality and introduces potential selection bias. Second, the 9‐year minimum follow‐up resulted in a 20.3% loss to follow‐up, comparable to other long‐term shoulder instability studies. This attrition may overestimate functional outcomes (e.g., ASES, Rowe scores), though sensitivity analyses showed no significant differences in baseline characteristics between followed and lost patients (*p* > 0.05). Third, the absence of a control group (e.g., open Latarjet with screw or plate fixation) precludes direct comparison of the MASB Latarjet with alternative techniques. However, outcomes of traditional Latarjet procedures (e.g., 10%–15% complication rates, 5%–10% arthritis at 10 years) reported in the literature provide a benchmark for indirect comparison. Finally, the Samilson–Prieto classification for glenohumeral arthritis may lack sensitivity for detecting early osteoarthritic changes, potentially underestimating subclinical degeneration. Future studies with randomized designs, comparative arms, and more sensitive imaging modalities (e.g., magnetic resonance imaging) are needed to address these limitations. As a comment on study design, the final cohort of 106 patients substantially exceeded the a priori target sample size of 42 (accounting for 20% attrition), providing robust statistical power to detect meaningful changes in ASES scores and other outcomes.

## CONCLUSION

The MASB Latarjet provided durable shoulder stability and high patient satisfaction at 9‐year follow‐up, with low complications, minimal ROM loss and no evident glenohumeral degeneration. These findings support its use as an effective alternative to traditional techniques, especially in centres with arthroscopic expertise.

## AUTHOR CONTRIBUTIONS

**Wei Lu**: Designed the project; interpreted the data; reviewed the manuscript. **Daqiang Liang**: Collected the data; reviewed the manuscript. **Shengwu Yang**: Data collection. **Yan Liu**: Data analysis. **Guoqing Cui**: Reviewed the manuscript. **Chunwu Zhang**: Reviewed the manuscript. **Shuguang Gao**: Designed the study and reviewed the manuscript. **Zhenhan Deng**: Designed the study; provided funding support; interpreted the data; wrote the manuscript.

## CONFLICT OF INTEREST STATEMENT

The authors declare no conflict of interest.

## ETHICS STATEMENT

The authors have nothing to report.

## Data Availability

The authors have nothing to report.
